# Medical Opinion and Movement

**Published:** 1908-03-07

**Authors:** 


### Medical Opinion and Movement.

PUBLIC health authorities in the United States have not been slow to grasp the importance of the definite pronouncements in India and Australia on the spread of plague by rats, and strenuous efforts are being made to exterminate the species, particularly in San Francisco, but also at Oakland Point, Richmond, and Emeryville, California. Statistics of the rats killed or found dead are published weekly. Of 1,371 rats examined bacteriologically nineteen were found to be infected with the bacillus pestis. The rats are trapped in large numbers. They are submitted to bacteriological examination, and laborious "flea censuses" are made by combing out and counting the fleas from previously chloroformed rats. The percentage of plague-infected rats is on the decrease, being about 0.7 per cent. of the number examined, and plague is practically at a standstill, no case having occurred in San Francisco since December 20. Stringent precautions are being taken to render buildings and stables rat-proof, and the use of metal garbage cans has been ordered. San Francisco has special reason to be concerned about the disease on account of its floating cosmopolitan population, and the rest of the civilised world has reason to be gratified at these active measures, as the city might easily form a dangerous focus of infection to other communities if it fell a prey to a serious outbreak of plague.

WITH the continual influx of new synthetic preparations there is a tendency to neglect old and well-tried remedies. This is to be regretted, for the hopes raised by the vendors of these new drugs in the elaborate pamphlets that usually accompany the samples are seldom realised, and but a very small proportion of these synthetic chemicals maintain a lasting reputation with the profession. Dr. Eustace Smith draws attention to these facts, and pleads for a rehabilitation of antimony into the working pharmacopœia of the general practitioner. Antimony has fallen into discredit on account of the depression which accompanies the administration of large doses. Its use in this way was associated with the antiphlogistic method of treatment of fevers and inflammatory conditions, and with the abandonment of this method antimony has shared a like fate. Dr. Eustace Smith claims that antimony in small doses is a drug of the utmost value, and that in catarrhal states of the mucous membrane it has never been dislodged from its eminence by later substitutes. He is of opinion that the younger generation of medical practitioners have been led into erroneous methods of treatment of bronchitis by giving in the earlier stages a combination of stimulating and antispasmodic drugs, such as ammonia, squill, and paregoric, which are calculated to make the cough harder and the chest tighter, and to aggravate the discomfort of the patient. These drugs should be reserved for the later stage of the disease, when the cough is loose, with a free secretion of mucus. In the early stages Dr. Smith advises a combination of vinum antimoniale in small doses, with diaphoretics such as acetate of ammonia and nitrate of potash. In broncho-pneumonia of children he finds antimony also of great value combined with belladonna. There are several other conditions, such as laryngismus stridulus, gastric disorders, and eczema, in which he finds the drug useful. Drugs have fashions, and there is no doubt that in recent years antimony has been out of fashion and its therapeutic value has probably been much undervalued.

DR. ALDO CASTELLANI, of Colombo, has recently reported his results of the treatment of experimental trypanosomiasis in monkeys. He was unable to obtain the trypanosome of sleeping sickness, but inoculated with a strain from a bullock of Indian breed, probably T. evansi. He used mercuric chloride injections intravenously, and also injections of quinine cacodylate. He found that the mercury perchloride caused a subsidence in the temperature, but it appeared to have only a feeble trypanocide action, and the improvement was only temporary. On the other hand, quinine cacodylate showed a well-marked trypanocide action in monkeys. After fifteen to twenty-five injections the trypanosomes were no longer present in the peripheral circulation nor in the enlarged glands, and the fever disappeared. Recurrences took place, however, in about 30 per cent. of cases, and it was found that the drug had a much weaker action then than in the first attack. This point is of considerable interest, as it offers support to Ehrlich's view that the trypanosomes may become partially or totally immune to the action of a drug when subjected to it for some time. A combined treatment of mercury perchloride and cacodylate of quinine appeared to give the best results in the treatment of monkeys. Dr. Castellani suggests that this combination should be tried in sleeping sickness. If it should be found that quinine cacodylate is efficacious in this disease there would be an additional advantage in its use on account of its powerful action on the malaria parasites. A large number of patients suffering from sleeping sickness are said to be also victims of chronic malaria. The same drug has also been found beneficial in kala-azar and in yaws.

THERE have been various speculations as to the possible toxic effects of burned petrol fumes, but hitherto little or no evidence has been recorded in support of any evil effects they may exercise upon the human subject. Sir William R. Gowers has, however, just published an account of a case which he terms pseudo-myasthenia of toxic origin, and which he traces to the continual exposure to these fumes of burned petrol. The patient was an officer in the Army, engaged in a Government factory, and superintending the construction and testing of petrol engines. The condition commenced with a perversion of the sense of taste, all sweet things having for him a salt taste. This was followed by a constricted feeling in the throat when swallowing. This feeling developed in the course of a few months to a definite difficulty of swallowing anything except bland fluids.

The voice then developed myasthenic characters, becoming feeble with imperfect articulation, especially after a few minutes' talking. The contractions of the obicularis palpebrarum muscles were very weak, and there was little outward movement of the mouth on smiling. The electrical reactions, reflexes, and sensation were normal. The symptoms presented a striking similarity to those of myasthenia gravis. The case suggested to Sir William Gowers the possibility of a toxic cause, and the fumes of burned petrol were the only source discoverable. The condition rapidly yielded to treatment with hypodermic injections of strychnine. After an interval of some months the patient was enabled to resume his work, but in the course of another year the symptoms again returned. Under the same treatment and with abstention from work recovery again rapidly took place, so that the causal connection appears to be pretty well proved. In view of the increasing use of petrol and our present ignorance as to the possible baneful effects of its fumes the case is one of considerable interest.

---

The subject of the care of children's teeth, and its importance in relation to national health and physique, which has lately been dwelt upon in the pages of The Hospital, forms the subject of a paper by an American dentist, Mr. C. C. Mottinger, in the *Dentists' Magazine*. The author urges that, since the instruction of the youths of the United States in the physiological and pathological effects of alcohol and narcotics has already met with remarkable success, the Government should extend this form of public school instruction to the care and preservation of the teeth. Children should no longer be encouraged to avoid the sight of a dentist's "office," but should gradually be educated to regard it as a place to be sought if health and beauty are to be enjoyed to their full. Mr. Mottinger rightly considers that the adoption of proper prophylactic methods is the crying need of the day.

---

Dr. Alfred de Roulet, of Chicago, in an article on the non-operative treatment of diseases of women in the *Dublin Journal of Medical Sciences*, makes a strong appeal for the medical, as opposed to the surgical, treatment of uterine displacements and the various chronic inflammations and congestions of the pelvic organs. He recommends a judicious combination of local and constitutional measures in the treatment of most gynæcological affections. Among other methods of local treatment, he discusses the value of electricity. This he considers of less use in the cure of pelvic disease than in the relief of pelvic pain. He advocates the use of the fine faradic coil for this purpose, one electrode being introduced into the vagina or uterus, the other externally over the fundus, and the current being passed for fifteen to thirty minutes every second or third day. The current should be turned on and off gradually. For intrauterine applications a bipolar electrode is preferable. Dr. de Roulet maintains that this treatment has a very good effect upon ovarian neuralgia and neurotic abdominal pains, as well as upon the pain of pelvic inflammations.

Dr. John Wyllie, in an article in *The Monthly Cyclopædia of Practical Medicine* on the serum-treatment of diphtheria, comes to the following conclusions, based upon his own practical experience:—He has no doubt that the hypodermic injection of antidiphtheritic serum shortens the first stage of the illness, preventing further formation of membrane and causing the rapid removal of that already formed, and thus greatly reducing the possibility of auto-infection; also that it makes the necessity for tracheotomy much less frequent than formerly. He further feels convinced that when tracheotomy is necessary it is much more likely to be successful when the patient has been placed under the influence of antidiphtheritic serum. Finally, Dr. Wyllie's opinion is that when so much has been said in favour of this antitoxin, everything has been said that can be advanced in its favour.

---

Grunmach and Bickel have put on record a recent case of "stone cough." The patient, a woman aged thirty-five, had enjoyed moderately good health up to eight years before she came under observation. At that period she had an attack of pleurisy, accompanied by fits of coughing. These fits persisted, with occasional remissions, and on two occasions the patient had severe attacks of hæmoptysis. At present she has two or three attacks during the year. The paroxysms are severe, and little sputum is expectorated; but the patient coughs up moderately sized concretions, irregular and apparently calcareous. No tubercle bacilli were found, but a screen examination revealed several large, irregular patches of calcification in the right lung. The concretions vary in size from that of a pea to a sand grain, and they are composed of epithelial debris, mucin, leucocytes, and calcareous particles.

---

During the past six and a half years Mr. James Berry has operated upon 268 patients for removal of goître. There are several interesting points in the report on these cases which he presented to the Royal Society of Medicine in its Surgical Section. In 274 operations his chief reason for operating was as follows:—Dyspnœa (in 177); deformity (33); discomfort or deformity, mostly with slight dyspnœa (47); malignancy, patent or suspected (13); dysphagia (1); and increasing size (3). The goître was encapsuled in 203 cases, non-encapsuled in 71; innocent in 267 cases, malignant in 7. Mr. Berry, as the outcome of his experience of 400 operations, considers that dyspnœa is never a contra-indication of removal, but is rather an important reason for operating. He believes that tracheotomy is bad treatment for the dyspnœa of cases that are not malignant. Dyspnœa in goître is, in his opinion, invariably due to direct pressure upon the trachea. A general anæsthetic may be given in most operations for removal of the goître. The patient should be in the semi-recumbent position, and the incision recommended by Mr. Berry is the usual curved one. Like most surgeons, he is very doubtful of the advisability of removal in true cases of Graves' Disease.

THE draft bill for "the regulation of the practice of medicine by unauthorised and incompetent persons" in Germany has just been published. It deals, *inter alia*, with the sale and purchase of secret and proprietary medicines, and its schedules and clauses are well worth the consideration of the profession in this country. Authority is given to the Federal Council by this bill to limit, or entirely to prohibit, the sale of secret remedies if their use is liable to cause injury to health, or if they are sold in such a manner as to deceive the purchaser. By section 5, subsection 3, the importation of such articles is made penal. Special sections deal with public announcements, advertisements and recommendations for the "treatment" of various diseases, with obscene and objectionable publications on quasi-medical subjects. Adequate penalties are prescribed. Thus the contravention of the prohibition clause renders the offender liable to imprisonment for a period not exceeding a year plus the imposition of a fine of £150, or to either of these penalties at the discretion of the court. The duties of preparing the schedules and advising the Federal Council as to the steps to be taken in any particular case will devolve upon a special sub-committee, which will co-operate with the Imperial Board of Health. Members of this sub-committee will be recruited from the medical, pharmaceutical and veterinary professions, and will be appointed for five years. The draft bill, which will be brought forward during the present session of the Reichstag, has the unanimous support of both the medical and pharmaceutical profession in Germany.

---

HECKMANN has published, in the last number of the *Post-Graduate*, an interesting *résumé* of a case of "pulled elbow." The lesion is one which is by no means uncommon in young children under five years of age, but its pathology has been very much discussed of late years. There is usually a history of strain exerted upon the injured arm, the child having been lifted up from the floor by the mother grasping both forearms, or having been suddenly pulled back by the arm to save it from falling. Almost immediately afterwards the arm hangs flaccid by the side, the forearm slightly flexed and pronated. Power of extension is impaired and of supination usually lost, and there is a good deal of pain. On examination nothing definite can be made out, there being no swelling, crepitus, nor signs of dislocation or fracture. The relations of the bony prominences at the elbow are preserved, but in some cases there may be pain on pressure over the head of the radius, and close examination may reveal a separation between the head and the humerus. The treatment is easy. The upper arm is steadied and the forearm is forcibly extended and supinated. This is generally quite sufficient to remove all the symptoms. The child being able, almost immediately afterwards, to use its arm properly. Goyraud, who first described this lesion, supposed that its real site was the inferior radio-ulnar articulation, and that it consisted in a subluxation of the interarticular fibro-cartilage. Streubel and Hult Krauzt agreed that the main lesion was a partial dislocation of the radial head; but other observers incline to believe that the lesion is caused by compression of the interosseous membrane between the two forearm bones during sudden pronation. Yet a fourth theory ascribes it to a jamming of the joint capsule at the humero-radial articulation. Heckmann, after a study of several cases, is inclined to support Streubel's view, namely that the lesion consists essentially of a subluxation of the radial head.

---

THE author rightly considers that the lesion and its treatment are not well known, and that cases of "Goyraud's accident" are overlooked by the general practitioner. Usually the case is regarded as fracture or as a simple "sprain" of the elbow, wrist, or even shoulder. In the circumstances the patient is allowed to suffer pain for many days, the treatment adopted being wholly unsuitable. The lesion occurs five times more commonly in the left arm, and the patients are usually girls. The author has had thirty-two cases, all of which were cured instantly by proper manipulation as described above. If extension and supination of the forearm are followed by a quick flexion, reduction is easier, and in many cases saves a repetition of the manipulation. Where the lesion has remained untreated for several days there will be some stiffness after reduction, but even in such a case the patient is able to use the forearm normally within a couple of hours.

---

SCHEPELMANN has found veronal of great benefit in the treatment of obstinate cases of sea-sickness. He counsels his patients to avoid the sides of the boat, to occupy themselves on deck as much as possible, and to avoid taking a heavy meal before embarking on a voyage. In inveterate cases he prescribes a mixture of hydrochlorate of cocaine, tincture of iodine, and water, to be taken as a draught. As a prophylactic he advises the use of veronal, 1 to 2 grammes being taken in the course of twenty-four hours. Where there is much distress and retching he administers the veronal in a milk enema, giving 1 gramme as a single dose. He states that he has most excellent results since adopting this method of treatment.

---

BECK of Chicago has been fortunate enough to obtain for examination the nasal septum of a man on whom he performed, two and a half years ago, the "window" operation for resection of the septal cartilage. Hitherto there has been much discussion as to how the cartilage is regenerated after such resection. Freer thought that the septum was repaired by actual regeneration of both bone and cartilage, and most histologists agree that cartilage is slowly regenerated conditionally upon the muco-perichondrium being preserved. Beck's patient died of pneumonia two and a half years after operation, and at the autopsy the septum was apparently quite firm. Microscopically sections of the septal tissue showed no trace of bone or cartilage, their place having been taken by a well organised layer of fibrous connective tissue, with a layer of normal mucous membrane on each side.

